# Enhancing Immunotherapy Response Prediction in Metastatic Lung Adenocarcinoma: Leveraging Shallow and Deep Learning with CT-Based Radiomics across Single and Multiple Tumor Sites

**DOI:** 10.3390/cancers16132491

**Published:** 2024-07-08

**Authors:** Cécile Masson-Grehaigne, Mathilde Lafon, Jean Palussière, Laura Leroy, Benjamin Bonhomme, Eva Jambon, Antoine Italiano, Sophie Cousin, Amandine Crombé

**Affiliations:** 1 Department of Diagnostic and Interventional Oncologic Imaging, Institut Bergonié, F-33076 Bordeaux, Francej.palussiere@bordeaux.unicancer.fr (J.P.); 2 Department of Radiology, Pellegrin University Hospital, F-33000 Bordeaux, France; eva.jambon@chu-bordeaux.fr; 3 Department of Medical Oncology, Institut Bergonié, F-33076 Bordeaux, Francea.italiano@bordeaux.unicancer.fr (A.I.);; 4 Department of Biopathology, Institut Bergonié, F-33076 Bordeaux, France; 5 SARCOTARGET Team, Bordeaux Institute of Oncology (BRIC) INSERM U1312, F-33076 Bordeaux, France

**Keywords:** lung adenocarcinoma, metastasis, radiomics, response evaluation, machine learning, deep learning

## Abstract

**Simple Summary:**

Efficient prognostic tools for predicting progression-free survival (PFS) in metastatic lung adenocarcinoma (MLUAD) patients undergoing first-line immunotherapy are lacking. This study aimed to enhance prognostic accuracy by leveraging advanced machine-learning survival models and single- and multi-site radiomics data extracted from pre-treatment CT scans, and comparing them to traditional clinicopathological data analyzed using a Cox regression model. Conducted retrospectively on a cohort of 140 patients treated at our comprehensive cancer center, the study revealed significant correlations between various radiomics-based features and PFS, particularly regarding those data extracted from the largest tumor lesion per patient and those summarizing the radiomics profiles of all tumors per patient, as well as the radiophenotypic divergence across all metastases within each patient. Notably, Deepsurv, incorporating carefully selected clinicopathological and radiomics-based inputs, and GBM, utilizing all input variables, demonstrated superior prognostic performance in repeated cross-validation. Additionally, the integration of radiomics with shallow- and deep-learning models surpassed the predictive ability of conventional Cox models, whatever their clinicopathological or radiomics inputs, thereby enhancing prognostic capabilities in MLUAD patients undergoing immunotherapy.

**Abstract:**

This study aimed to evaluate the potential of pre-treatment CT-based radiomics features (RFs) derived from single and multiple tumor sites, and state-of-the-art machine-learning survival algorithms, in predicting progression-free survival (PFS) for patients with metastatic lung adenocarcinoma (MLUAD) receiving first-line treatment including immune checkpoint inhibitors (CPIs). To do so, all adults with newly diagnosed MLUAD, pre-treatment contrast-enhanced CT scan, and performance status ≤ 2 who were treated at our cancer center with first-line CPI between November 2016 and November 2022 were included. RFs were extracted from all measurable lesions with a volume ≥ 1 cm^3^ on the CT scan. To capture intra- and inter-tumor heterogeneity, RFs from the largest tumor of each patient, as well as lowest, highest, and average RF values over all lesions per patient were collected. Intra-patient inter-tumor heterogeneity metrics were calculated to measure the similarity between each patient lesions. After filtering predictors with univariable Cox *p* < 0.100 and analyzing their correlations, five survival machine-learning algorithms (stepwise Cox regression [SCR], LASSO Cox regression, random survival forests, gradient boosted machine [GBM], and deep learning [Deepsurv]) were trained in 100-times repeated 5-fold cross-validation (rCV) to predict PFS on three inputs: (i) clinicopathological variables, (ii) all radiomics-based and clinicopathological (full input), and (iii) uncorrelated radiomics-based and clinicopathological variables (uncorrelated input). The Models’ performances were evaluated using the concordance index (c-index). Overall, 140 patients were included (median age: 62.5 years, 36.4% women). In rCV, the highest c-index was reached with Deepsurv (c-index = 0.631, 95%CI = 0.625–0.647), followed by GBM (c-index = 0.603, 95%CI = 0.557–0.646), significantly outperforming standard SCR whatever its input (c-index range: 0.560–0.570, all *p* < 0.0001). Thus, single- and multi-site pre-treatment radiomics data provide valuable prognostic information for predicting PFS in MLUAD patients undergoing first-line CPI treatment when analyzed with advanced machine-learning survival algorithms.

## 1. Introduction

Non-small cell lung cancer (NSCLC) is the first cause of cancer mortality, with 1.8 million deaths worldwide in 2020, despite the rise of precision oncology [1]. Lung adenocarcinoma represents its most frequent histological subtype, with 57% of all new cases and nearly half of patients being diagnosed with metastases [2]. Importantly, the majority of deaths occur in metastatic patients, making imperative a better comprehension of the metastatic stage [1].

In this advanced setting, the guidelines from the European Society of the Medical Oncology and the American Society of Clinical Oncology recommend to assess programmed-death ligand 1 (PD-L1) status and a panel of targetable molecular alterations in order to decide the first-line systemic treatment among tyrosine-kinase inhibitors (TKIs), immune checkpoint inhibitors (CPIs), and platinum-based chemotherapy [3]. Although CPIs have revolutionized the managements and outcome of metastatic lung adenocarcinoma (MLUAD) patients, biomarkers of the treatment response are crucially lacking. Indeed, the 5-year survival rate of patients with a PD-L1 tumor proportion score (TPS) ≥ 50% is 29.6%, despite appropriate treatment with CPI [4].

Various research directions are being explored to better identify patients who would benefit from CPI (and to avoid inefficient, costly, and potentially toxic treatments), from genomic alterations [5,6], corrected tumor mutational burden [5], tertiary lymphoid structures, and other immunohistochemistry panels of the tumor microenvironment [7], to imaging.

Contrast-enhanced computed tomography (CT scan) is the commonest and best-recommended imaging modality in MLUAD. However, the potential information contained in a CT scan is strongly underexploited and is summarized to the tumor location and size, despite marked heterogeneity of the disease presentation on imaging, between patients, between tumor lesions from a same patient, and within a same lesion, which could be estimated with radiomics. Radiomics correspond to the extensive quantification of the radiological phenotypes (or radiophenotypes) of cancers on any imaging modality (including CT scan), beyond what radiologists can describe with verbal descriptors [8,9,10]. Radiomics relies on the calculation of hundreds of numeric descriptors, named radiomics features (RFs), which quantify the texture and shape of the segmented tumors. The RFs are then mostly mined in supervised machine-learning algorithms in order to create predictive signatures for relevant oncologic outcomes, including the response to treatment and progression-free survival (PFS). Regarding NSCLC response to CPI, several radiomics studies have demonstrated significant associations between single-site radiomics and response [11,12,13,14,15].

However, the application of radiomics approaches in metastatic patients remains understudied, representing a significant gap in our understanding of the disease. In addition, the management of several potential and correlated potential predictors in a highly multidimensional radiomics dataset can be critical for traditional survival algorithms such as the Cox proportional hazards regression [16], which assumes a simple linear relationships between the features. Hence, innovative survival algorithms have been specifically designed to capture more complex and possibly non-linear relationships between the input variables, including least absolute shrinkage and selection operator (LASSO) penalization for Cox regression [17], random survival forests (RSF) [18], gradient boosting machines (GBM) [19,20], and deep learning, notably, Deepsurv [21,22].

Hence, our main objective was to assess the potential of (i) pre-treatment CT-based radiomics derived from single and multiple tumor sites and (ii) advanced machine-learning survival algorithms, in predicting PFS patients with MLUAD undergoing treatment with CPIs as first-line treatment.

## 2. Materials and Methods

### 2.1. Study Design

This retrospective, single-center observational study was approved by the institutional review board of Bergonié Institute (regional comprehensive cancer of Bordeaux, France) in agreement with good clinical practice and applicable laws. Written consent was not considered necessary by the review committee due to the retrospective nature of the study. All research procedures and protocols adhered to the principles set forth in the Declaration of Helsinki.

All consecutive adult patients with newly diagnosed, histologically proven lung adenocarcinomas between November 2016 and November 2022 were included as they filled the following inclusion criteria: available whole-body contrast-enhanced CT scan, patients with metastatic disease at baseline, presence of at least two measurable target lesions (according to the response evaluation criteria in solid tumors [RECIST] v1.1) with volume ≥ 1 cm^3^, and entire therapeutic management in our comprehensive cancer center with 1st line treatment comprising CPI (alone or combined with platinum-based chemotherapy) [23].

Exclusion criteria were the following: other concomitant cancer, no pre-treatment routine molecular screening or PD-L1 status, poor quality CT scan (i.e., artifacts on target lesions), patients with significantly altered clinical state at diagnosis (i.e., World Health Organization performance status [WHO-PS] ≥ 3), and patients who died before starting treatment.

**Figure 1:**
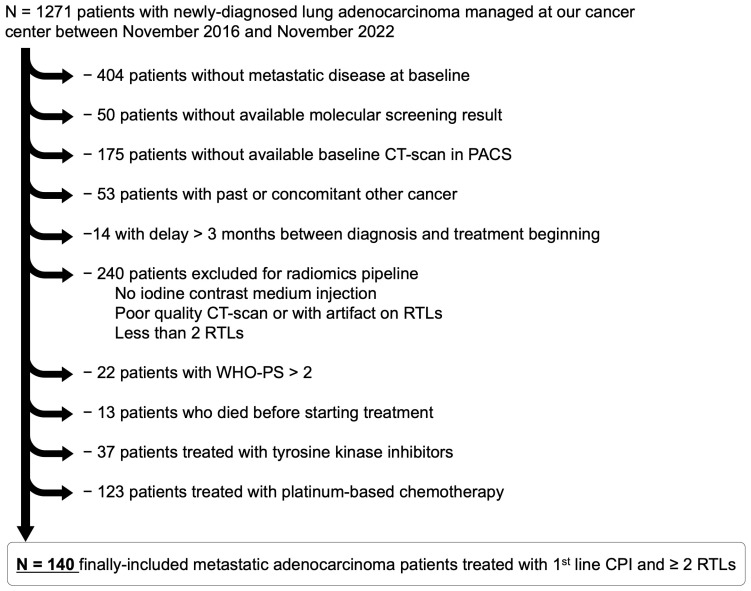
Study flowchart. Abbreviation: CPI, checkpoint inhibitor; CT, computed tomography; PACS, picture archiving and communication system; RTL, radiomics target lesion; WHO-PS, World Health Organization performance status.

Figure 1 shows the study flow chart.

The main outcome was the PFS, defined as the time (in months) elapsed from the 1st day of treatment to the date of progression or death related to disease, or last follow-up. Patients lost to follow-up at the end of the data collection (February 2024) or dead for a reason unrelated to cancer were censored. Progressive disease was assessed on routine revaluation CT scans performed every 3 months according to the RECIST v1.1 criteria by senior radiologists from our cancer center.

### 2.2. Data Collection

#### 2.2.1. Clinical Data

The following data were collected from medical records: age (in years, and further categorized as < or ≥70 years), sex, tobacco addiction, WHO-PS, initial staging, location of metastases, presence of brain, liver and bone metastases, number of distinct metastatic locations (categorized as 1, 2, 3, and ≥4), and type, if first-line treatment (categorized as CPI or chemotherapy + CPI).

#### 2.2.2. Pathological and Molecular Data

The following data were collected from the pathological analyses of the pre-treatment biopsies: programmed-death ligand 1 (PD-L1) status, categorized as 0%, 1–49%, and 50–100% depending on the immune-histochemical tumor positive score [24,25]. Of note, patients with a PD-L1 status of 0% were included, as the associations between CPI and chemotherapy (pemetrexed + platinum-based regimens) were recommended in non-squamous NSCLC without targetable alterations in good performance status whatever the PD-L1 status [26,27].

Molecular screening was obtained on a routine basis using next-generation sequencing analysis on pre-treatment tumor sample according to panels recommended by the French guidelines [28] and comprising KRAS, EGFR, ALK, ROS1, RET, TP53, PI3K, STK11, MET, BRAF, and HER2. Patients with EGFR, ALK, ROS1, and RET alterations were inherently excluded from the study, as they received tyrosine kinase inhibitor at first line, however, the number of identified alterations and the presence of KRAS and TP53 alterations were collected.

### 2.3. Radiomics Workflow (Figure 2)

#### 2.3.1. CT Scan Post-Processing

Contrast-enhanced CT scan covering the brain, thorax, abdomen, and pelvis in the abdominal kernel were first pseudonymized and converted from the DICOM to the nifti format using the dcm2niix free converter (github.com/rordenlab/dcm2niix accessed on 2 December 2023). CT scans were then exported to the LIFEx freeware (v7.1.17, Saclay, France), which is compliant with the Imaging Biomarker Standardization Initiative (IBSI) [29,30]. Two radiologists (C.M.G and A.C. with 2 years and 10 years of experience in oncologic imaging, respectively), blinded to all clinical data, manually segmented in 3D, slice-by-slice, all the tumor lesions in each patient, as long as they filled the criteria for a measurable solid lesion per RECIST v1.1 and demonstrated a volume > 1 cm^3^—which defined radiomics target lesions (RTLs) [23]. For bone metastases, only the extra-osseous tissue component was selected. For lung lesions, excavations were avoided. One hundred RTLs were randomly selected and eroded by one voxel in order to obtain a second volume-of-interest (VOI-eroded, in addition to the initial VOI) and estimate the inter-segmentation reproducibility of the radiomics features (RFs), according to good radiomics practices [9]. The segmentations performed by the youngest radiologists were all verified by the senior radiologists. Moreover, the radiologists encoded the location and longest diameter of each RTL.

**Figure 2:**
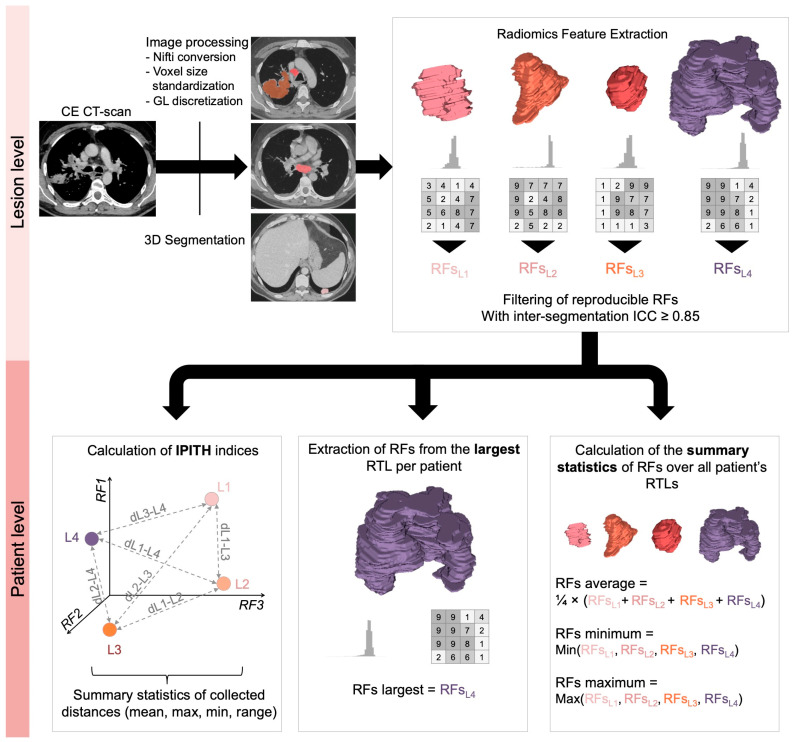
Radiomics workflow. Abbreviations: dLx-Ly, distance between lesion x and lesion y; RF, radiomics features; RTL, radiomics target lesion; IPITH, intra-patient inter-tumor heterogeneity. Regarding the calculation of IPITH metrics, the related scheme has been simplified to a representation of the lesions in a radiomics space of 3 dimensions (i.e., 3 reproducible RFs) to facilitate its understanding, but it actually corresponded to 68 RFs.

#### 2.3.2. Radiomics Features Extraction

After analyzing the distribution of the raw Hounsfield units (HUs) contained in all the RTLs, the densities were discretized into 120 gray levels of a width of 2.5 HUs from −100 HU to +200 HU (higher and lower values were excluded from the RF calculations). The voxel sizes were all standardized to a common size of 1 × 1 × 2 mm^3^ using b-spline interpolation. Afterwards, 121 RFs were calculated in 3D (definition and formula are given at lifexsoft.org/index.php/resources/documentation accessed on 2 January 2024) [29]. Gray-level co-occurrence matrix (GLCM) features were computed in 13 directions and for one voxel displacement.

#### 2.3.3. Radiomics Features Filtering and Transformation

The RFs were calculated a second time for the 100 eroded VOIs, which enabled to calculate intra-class correlation coefficient (ICC) in order to estimate the robustness of the 121 RFs to perturbations in the segmentation. Only RFs with ICC > 0.85 were selected for the remaining of the analyses. In addition, RFs with near zero variance in the whole cohort were excluded. Hence, 68 RFs were finally included in the study (10 shape RFs, 26 histogram-based RFs, and 32 s order RFs), detailed in Supplementary Table S1. Next, RFs were center scaled to reduce their impact on distance measurements and on statistical modeling.

#### 2.3.4. Summary Statistics Based on Radiomics Features

First, as most radiomics models in the oncologic imaging literature rely on a single lesion, we extracted the RFs from the largest lesion in terms of tumor volume of each patient, whatever its location, which provided 68 largest-RFs.

Second, for each patient, we calculated the minimal, average, and maximal values of each RF over all its tumor lesions, which provided 68 average RFs, 68 minimal RFs, and 68 maximal RFs per patient.

#### 2.3.5. Quantification of Intra-Patient Inter-Tumoral Lesion Heterogeneity Using RFs

To measure the radiophenotypic dissimilarity (or distance) from one tumor lesion to one another in each patient in terms of shape and texture, we developed the following method based on radiomics (Figure 2). Briefly, each lesion can be represented as a point in the ‘radiomics space’ (with as many dimensions as robust radiomics features, i.e., n = 68 dimensions). Hence, for each patient, we calculated the distance between each pair of tumor lesions in this radiomics space, which enabled to obtain a vector of distances between lesions. Various distances have been described in the bioinformatics and machine-learning literature, depending on their performances on non-normal data, their sensitivity to outliers, negative data, scales or highly correlated dimensions; thus, we explored the following distances: Euclidean, Spearman, Jaccard, Minkowski, Canberra, and Chebyshev (rationale and formula are given in Supplementary Table S2) [31]. The last step consisted in summarizing this vector of distances per patient with classical descriptive statistics: mean, minimum, maximum, and range (which were also center scaled).

### 2.4. Statistical Analysis

**Figure 3:**
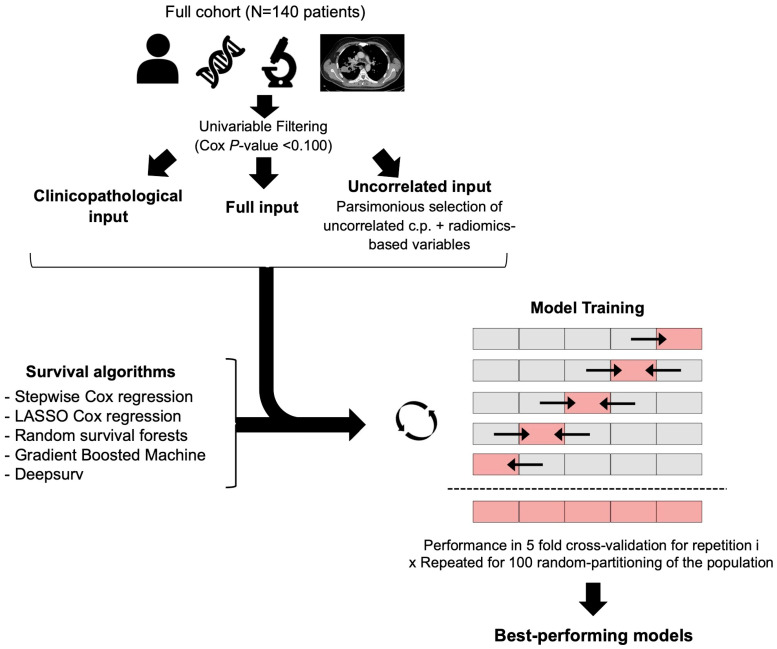
Statistical workflow. Survival algorithms were trained and evaluated according to concordance index using 100-times repeated 5-fold cross-validation and based on three types of inputs that were pre-filtered using univariable Cox regressions: (i) clinicopathological features, (ii) all radiomics-based and clinicopathological features, and (iii) uncorrelated parsimonious radiomics-based and clinicopathological features. Arrows indicate that in each fold of the 5-fold cross-validation, the models were trained on the 4 gray blocks and then applied and evaluated on the remaining light red block Abbreviations: c.p., clincopathological; LASSO, least absolute shrinkage and selection operator.

Statistical analyses were performed with R (The R foundation for statistical computing, v4.1.0, Vienna, Austria). All tests were two-tailed. A *p*-value less than 0.05 was deemed significant. The list of packages and functions used are provided in Supplementary Table S3. The overall statistical workflow is represented on Figure 3. Patients with any missing input variable were excluded from the multivariable analyses.

#### 2.4.1. Descriptive Statistics

All numeric variables were described as mean ± standard deviation, and median with 1st and 3rd quartiles and minimum-maximum range. Categorical variables were described as numbers and percentages.

#### 2.4.2. Univariable Survival Analysis

First, survival analyses were performed using the log-rank test for categorical variables and univariable Cox regression for both numeric and categorical variables to compute hazard ratios (HRs) and their 95% confidence intervals (95%CI). Clinicopathological and radiomics variables associated with a Cox *p*-value < 0.100 were filtered and selected for subsequent multivariable analyses (named ‘full input’).

Next, we assessed their pairwise correlations between all variables associated with PFS with *p* < 0.100 and only selected those without redundant information (i.e., Spearman test *p*-value > 0.05). In case of significant correlation, we excluded the variable with the highest univariable *p*-value (for pairs of RFs), or the most complex variable to obtain (i.e., as clinicopathological variables reflecting the tumor burden and metastatic spreading were easier to access compared to radiomics; they were systematically privileged over RFs). This step provided the ‘uncorrelated input’.

#### 2.4.3. Multivariable Survival Modeling

Five multivariable survival models were trained in 100-times repeated 5-fold cross-validation (rCV) to predict PFS under 1st line CPI with three types of input variables: (i) clinicopathological variables with univariable *p* < 0.100, (ii) full input, and (iii) uncorrelated input. The rate of progression was the same in each partitioning. In other words, for each of the 100 random repetitions of the rCV, the algorithm was trained on 4 of the folds (i.e., 112 patients) and evaluated on the remaining fold (i.e., 28 independent patients), repeating this process 5 times. This provides 5 performance estimates per repetition, which were then averaged over the folds and repetitions to reduce the variability and provide a more robust assessment of the model’s effectiveness.

Regarding hyperparameter selection, the tuning grids screened for all algorithms are given in Supplementary Table S4. The performance metric used for model selection was the Harrell concordance index (c-index), which ranges from 0 (worst possible) to 1 (perfect model) with 0.5 indicating a random model. C-indices were compared over the 100 repetitions.

- Stepwise Cox regression (SCR). This popular semi-parametric algorithm was used to benchmark more complex models. It assumes that the HRs are constant over time and the risks of experiencing an event are proportional over time for each level of the predictor variables (with a certain weighting) [16]. Herein, a stepwise backward process was added, based on the minimization of the Akaike information criterion, in order to select the final variables included in the model [32].
- LASSO Cox regression. This variation of the Cox regression includes a penalty term (i.e., the λ hyperparameter) to perform variable selection and regularization, which forces some coefficients to shrink towards zero and leads to a more parsimonious model [17].
- RSF. In this extension of random forests, multiple decision trees are created from a random bootstrapped subset of the training data and a random subset of predictors. At each split node of each tree, the algorithm selects the best split among the randomly selected predictors considering the time-to-event information (herein, according to log-rank score). After training, the predicted survival function for each patient is obtained by averaging the survival functions predicted by all trees in the forest [18]. The hyperparameters investigated in this work were: the number of variables to possibly split at each node (mtry) and the minimum size of terminal node (nodesize). The number of trees was set to 1000 and the splitting criterion to “log-rank”.
- GBM. In this extension of gradient boosting machines, the model is built by combining multiple decisions trees sequentially and iteratively (instead of independently, as in RSF), with each tree attempting to correct the errors made by the previous tree. A Cox’s partial likelihood loss function is used to measure the difference between the predicted and observed survival times and to optimize the model at each iteration (i.e., to decrease the prediction error). Moreover, a regularization is applied to limit the complexity of individual trees. Finally, after training, the predicted survival function for each patient is also obtained by combining the predictions from all trees in the ensemble. The hyperparameters investigated comprised the interaction depth (i.e., the highest level of variable interactions allowed), the learning rate, and the minimum member of observations in the terminal nodes of the trees. The number of trees was set to 1000 [19,20].
- Deepsurv. This recent deep-learning algorithm utilizes a multi-layer feed-forward neural network architecture to predict the hazard function from the input variables. Theoretically, it can learn complex and non-linear relationships between highly correlated covariates and survival times thanks to the optimization of a negative log partial likelihood Cox proportional hazards-based loss function and a gradient descent-based algorithm [21]. The hyperparameters investigated comprised the activation function, the optimizer, the number of hidden layers, and the number of nodes per layer. The number of epochs was set to 512 with early stopping to limit unneeded training, the batch size to 32 with a batch normalization, the momentum to 0.85, the learning rate to 0.01 with a learning rate decay of 0.001, the regularization to 15, and the drop out to 0.1, similar to the hyperparameters found in clinical datasets [21].

Of note, Deepsurv and LASSO Cox regression required one-hot-encoding of the categorical variables before training.

#### 2.4.4. Visualization and Understanding

For the best shallow- and deep-learning survival models, as well as the stepwise Cox model with clinicopathological variables (i.e., reference model), the time-dependent Brier score (BS), which measures prediction errors at a given time point, was calculated every 3 months from 0 to 2 years after treatment beginning with the same rCV partitioning, which enabled to represent the prediction error curves and calculate integrated Brier score (IBS) [33]. While the c-index evaluates a model’s ability to correctly rank the survival times (i.e., this is an assessment of the model’s discrimination power), the IBS evaluates the accuracy of the predicted survival probabilities over time. The IBS combines both discrimination and calibration. Therefore, displaying both c-indices and IBS enables to obtain a more comprehensive understanding of the best models’ performances. Kaplan–Meier curves for PFS for the relevant input variables were plotted. To understand the contribution of each predictor variables in the best-performing shallow- and deep-learning models, the importance of each input variable was estimated thanks to the permutation method with 100 permutations. To do so, for each predictor variable, the initial model’s performance was calculated, then the loss in performance was calculated after a random shuffling of its values, which was repeated 100 times and then averaged. The importances were then scaled for a total of 100% [34].

## 3. Results

### 3.1. Study Population (Table 1)

A total of 140 patients were finally included, with a median age of 65.2 years (Q1–Q3: 59.1–70.2, range: 42.5–87.9) and 36.4% (51/140) women. Regarding the first line, 30/140 (21.4%) patients were treated with CPI alone and 110/140 (78.6%) with CPI and platinum-based chemotherapy.

**Table 1:** Characteristics of the study population.

Characteristics	Patients (N = 140, with 663 RTLs)
**Sex**	
Women	51/140 (36.4)
Men	89/140 (63.6)
**Age (years)**	
Mean ± SD	64.26 ± 8.839
Median [Q1–Q3] (range)	65.2 [59.1–70.225] (42.5–87.9)
**WHO-PS**	
PS = 0	38/140 (27.1)
PS = 1	77/140 (55)
PS = 2	25/140 (17.9)
**Tobacco addiction**	
Never smoker	6/140 (4.3)
Active smoker	67/140 (47.9)
Former smoker	67/140 (47.9)
**Initial staging**	
IIIB-IVA	36/140 (25.7)
IVB	104/140 (74.3)
**PDL1**	
0%	43/140 (30.7)
1–49%	35/140 (25)
50–100%	62/140 (44.3)
**No. of altered genes on routine screening**	
0	29/140 (20.7)
1	73/140 (52.1)
≥2	38/140 (27.1)
**TP53 alteration**	
Yes	55/140 (39.3)
No or non-contributive	85/140 (60.7)
**KRAS alteration**	
Yes	67/140 (47.9)
No or non-contributive	73/140 (52.1)
**No. of distinct metastatic sites**	
1	35/140 (25)
2	36/140 (25.7)
3	29/140 (20.7)
≥4	40/140 (28.6)
**Bone metastasis**	
No	69/140 (49.3)
Yes	71/140 (50.7)
**Brain metastasis**	
No	108/140 (77.1)
Yes	32/140 (22.9)
**Liver metastasis**	
No	112/140 (80)
Yes	28/140 (20)
**No. of RTLs**	
Mean ± SD	4.7 ± 2.7
Median [Q1–Q3] (range)	4 [3–6] (2–15)
**Size of RTLs (mm)**	
Mean ± SD	30 ± 18
Median [Q1–Q3] (range)	23 [18–35] (10–144)
**Locations of RTLs**	
Abdominal carcinosis	33/663 (5)
Abdominal viscera	118/663 (17.8)
Bone	31/663 (4.7)
Brain	19/663 (2.9)
Lung	141/663 (21.3)
Lymph node	294/663 (44.3)
Pleura and pericardium	10/663 (1.5)
Soft tissue	17/663 (2.6)
**First-line treatment**	
CPI + Chemotherapy	110/140 (78.6)
CPI alone	30/140 (21.4)

NOTE—Data are numbers of patients with percentages for categorical variables, and mean ± SD and median [1st quartile [Q1]–3rd quartile range [Q3]] (minimum–maximum range) for numeric variables. Other abbreviations: CPI, checkpoint inhibitor; no., number; RTL, radiomics target lesion; SD, standard deviation; WHO-PS, World Health Organization performance status.

Regarding radiomics, 663 tumors were segmented, providing a median number of four RTLs per patient of (Q1–Q3: 3–6, range: 2–15), with the most frequent sites being lymph nodes (294/663, 44.3%), then lung (141/663, 21.3%), then adrenals (61/663, 9.2%). The average diameter of the RTLs was 30 ± 18 mm.

There were 116/140 (82.9%) progressions during the first line. The median PFS time was 6 months (95%CI: 5–9.4). The PFS probability at 2 years was 21.9% (95%CI: 15.9–30.2). Sixty-four patients (64/140, 45.7%) achieved an objective response during this first line.

### 3.2. Univariable Assessment

Univariable analysis for clinicopathological variables was performed. The univariable survival analysis for the clinicopathological variables is shown on Table 2. Three variables were associated with lower PFS: WHO-PS = 2 (HR = 2.37, 95%CI = 1.39–4.05, *p* = 0.0015—with WHO-PS = 0 as reference), ≥4 distinct metastatic sites (HR = 1.66, 95%CI = 1.01–2.74, *p* = 0.0462—with 1 metastatic site as reference), and presence of bone metastases at diagnosis (HR = 1.46, 95%CI = 1.01–2.11, *p* = 0.0439). Two variables demonstrated univariable *p*-values between 0.100 and 0.050, namely, initial staging (*p* = 0.0844) and PD-L1 status (*p* = 0.0825), and were also selected for multivariable analyses.

Univariable analysis for radiomics-based features was performed. Table 3 shows the 14 robust RFs and IPITH metrics that were correlated with PFS with *p*-value threshold of 0.100. The features from the largest tumor and the maximum RF values per patient were the most important contributors, with three and five selected ones, respectively (*p*-value range: 0.0076–0.0495). Regarding IPITH, the Canberra distance measurement provided significant results through the Canberra range (*p* = 0.0049) and Canberra-mean (*p* = 0.0006).

**Figure 4:**
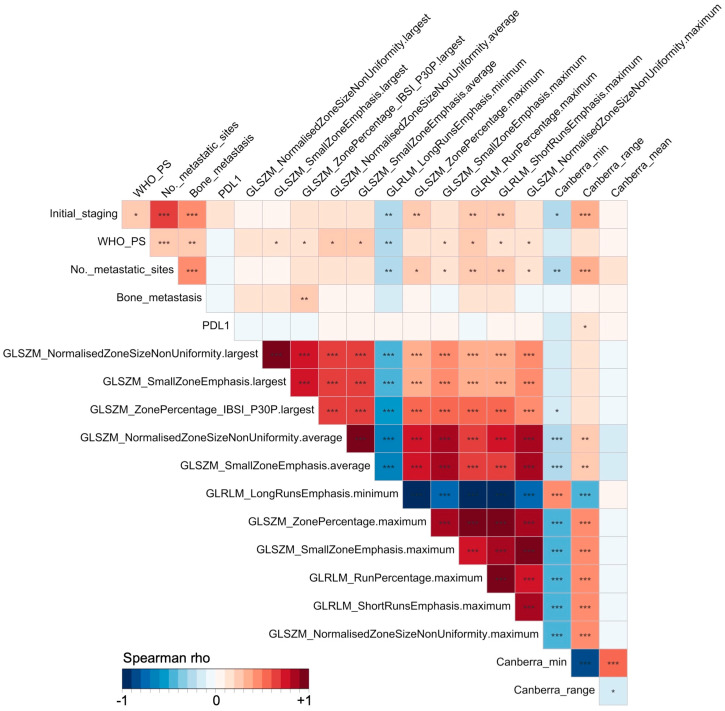
Correlation matrix of all the predictor variables tested in the multivariable survival modeling. Tests were non-parametric Spearman rank tests, with color-encoding of the Spearman rho value between −1 (blue, perfect negative correlation) to +1 (red, perfect positive correlation). Significance of the correlation tests is encoded as follows: *: *p* < 0.05, **: *p* < 0.005, ***: *p* < 0.001.

An assessment of correlations between the potential predictors was conducted. Figure 4 shows the correlations between the five clinicopathological features (i.e., clinicopathological input) and the 14 radiomics-based features (i.e., the full input of 19 variables) using non-parametric Spearman rank tests. After iteratively excluding the RFs and IPITHs with high correlation but a powerless association, or one more complex to obtain than the clinicopathological subset of variables reflecting the tumor burden, a smaller number were selected (i.e., the non-correlated input), namely, GLSZM_NormalisedZoneSizeNonUniformity from the largest tumor per patient and the Canberra-mean (i.e., uncorrelated input of seven variables [five clinicopathological and two radiomics-based]).

### 3.3. Performances of Survival Models in 100-Times Repeated 5-Fold Cross-Validation

Table 4 shows the c-index in rCV for each survival algorithm and for each type of input.

The highest c-index was obtained with Deepsurv trained on uncorrelated input (c-index = 0.631, 95%CI = 0.625–0.647), which was significantly higher than Deepsurv trained on clinicopathological input (c-index = 0.622, 95%CI = 0.602–0.647, *p* < 0.0001) and Deepsurv trained on full input (c-index = 0.613, 95%CI = 0.581–0.634, *p* < 0.0001).

Regarding shallow-learning algorithms, the best performing was GBM on the full input (c-index = 0.603, 95%CI = 0.557–0.646), which remained significantly lower than Deepsurv on the non-correlated input (*p* < 0.0001). It was closely followed by RSF on uncorrelated input (c-index = 0.602, 95%CI = 0.576–0.626).

**Figure 5:**
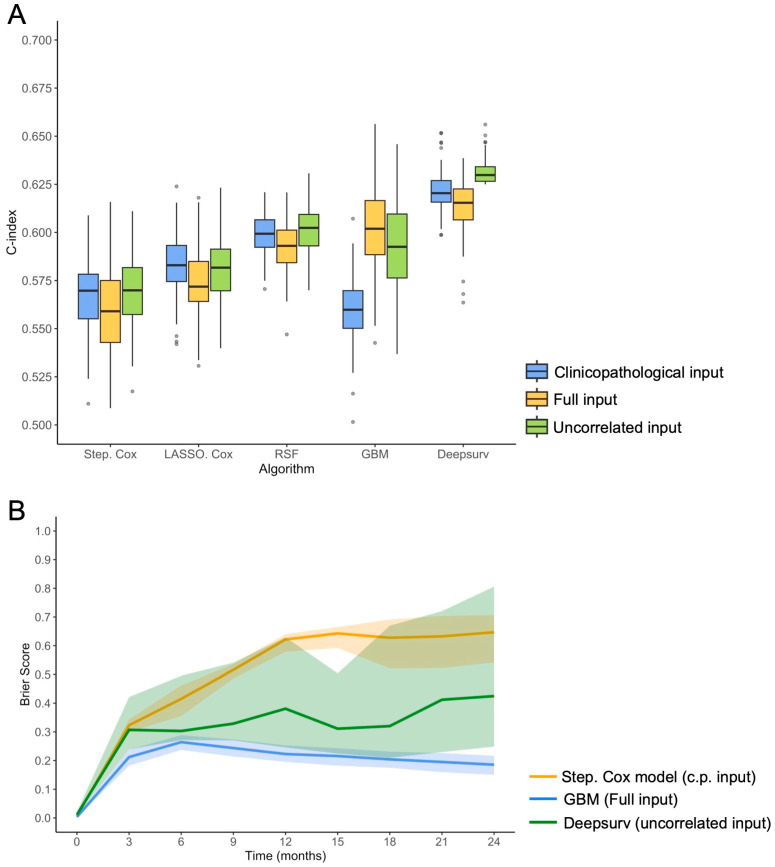
Survival models performance. (**A**) Boxplot of the concordance index (c-index) in 100-times repeated 5-fold cross validation, depending on the survival algorithm and the predictor variables initially entered in the modeling. Other abbreviations: Step. Cox, stepwise Cox regression; GBM, gradient boosted machine; LASSO, least absolute shrinkage and selection operator Cox regression; RSF, random survival model. (**B**) Brier score (with 95% confidence internal) in 100-times repeated 5-fold cross validation as a function of time (i.e., prediction error curve), between immunotherapy beginning and 2 years, for the benchmark model (stepwise Cox regression on the clinicopatholologic [c.p.] input), the best-performing shallow-learning model (GBM on full input), and the best-performing deep-learning model (Deepsurv on uncorrelated input).

Regarding the benchmark model, the c-index of the stepwise Cox regression on the clinicopathological input was 0.566 (95%CI = 0.525–0.601), which was significantly lower than Deepsurv on uncorrelated input (*p* < 0.0001) and GBM on full input (*p* < 0.0001). Supplementary Table S5 details the performances of usual stepwise Cox regression models on the entire cohort depending on the three types of input. Figure 5A represents the c-indices over the 100 repetitions of the rCV for all models and inputs. Figure 5B represents the average prediction error curves (with 95%CI) for the first 2 years for the Deepsurv model on uncorrelated input (rCV IBS = 0.322, 95%CI = 0.227–0.549), GBM on full input (rCV IBS = 0.207, 95%CI = 0.178–0.233) and the benchmark model (rCV IBS = 0.513, 95%CI = 0.454–0.550).

### 3.4. Understanding the Best-Performing Models

**Figure 6:**
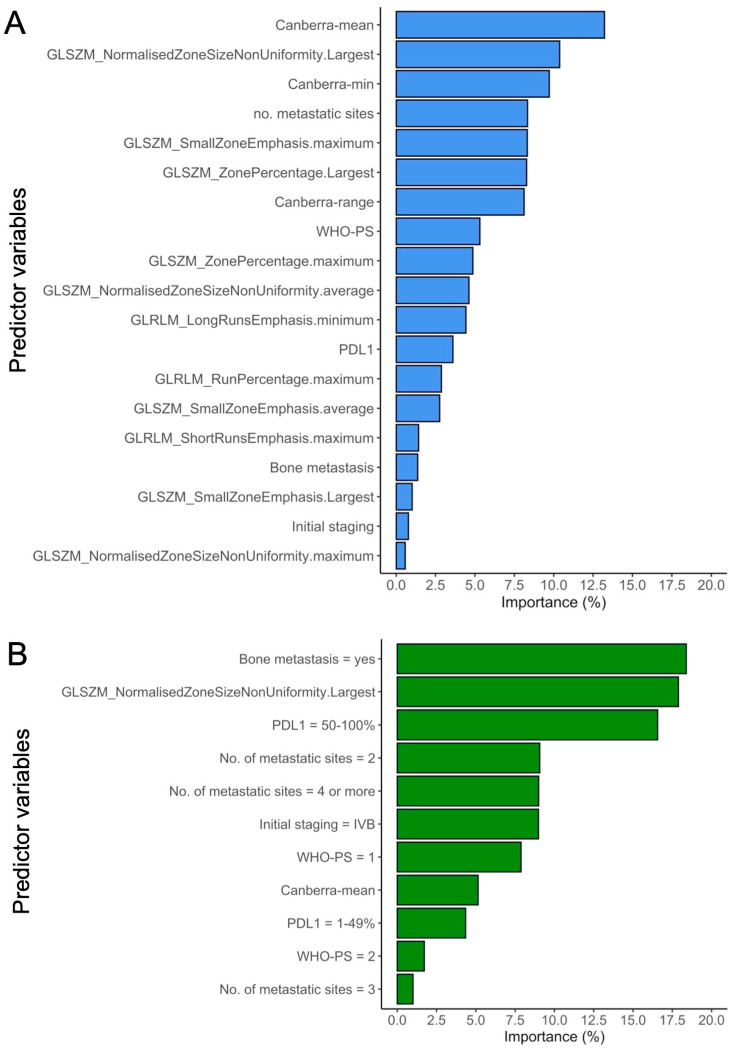
Importance of the predictor variables in the best performing models, i.e., Gradient Boosted Machine on all input variables (**A**) and Deepsurv of the uncorrelated input (**B**).

Figure 6 represents the most important variables in the GBM model utilizing the full input, and in the Deepsurv model based on the uncorrelated input.

In the Deepsurv best model, the most important feature was the presence of bone metastasis (18.4%), followed by GLSZM_NormalizedZoneSizeNonUniformity from the largest tumor site (17.9%) and PD-L1 TPS ≥ 50% (16.6%). The Canberra-mean was at the eighth position out of 11 (5.1%).

In the GBM best model, the most important feature was the Canberra-mean (13.2%), followed by GLSZM_NormalizedZoneSizeNonUniformity from the largest tumor site (10.4%) and the Canberra-min (9.7%).

## 4. Discussion

In this retrospective cohort of 140 patients with newly diagnosed MLUAD in a preserved health status, we explored (i) three sets of radiomics-based predictors alongside clinicopathological features, and (ii) five survival machine-learning models employing different designs with varying levels of complexity, aiming to predict the response to CPI given as first-line treatment. Our investigation revealed that integrating advanced shallow- and deep-learning models (GBM and Deepsurv, respectively) with carefully selected radiomics-based and clinicopathological predictors notably enhanced traditional Cox modeling.

CPIs, either alone or in combination with chemotherapy, have emerged as the first-line standard of care for patients with MLUAD without targetable alterations in EGFR, ALK, ROS1, or RET genes [3]. However, response rates to CPI treatment in unselected NSCLC patients typically fall between 43% and 49% for first line, and 15% and 20% for more advanced diseases [35,36,37], underscoring the critical need for biomarkers capable of predicting treatment sensitivity. This is essential to minimize treatment delays and prevent severe adverse events in non-responsive patients. Overall, developing an efficient model to predict PFS in MLUAD patients treated with CPI would be useful to better tailor treatments with more aggressive or alternative therapies in patients with high probabilities of not responding to CPI, and, consequently, to optimize patient outcome. Moreover, predicting PFS accurately could lead to more cost-effective treatment strategies.

Prior studies have suggested that artificial intelligence and radiomics hold promise in addressing this challenge [38]. Herein, we presented a comprehensive and pragmatic approach that incorporates (i) simple clinicopathological variables alongside complex numeric features quantifying intra-tumoral and inter-metastatic heterogeneity within patients, (ii) a range of survival models from simple Cox regression to advanced machine-learning techniques, and (iii) repeated cross-validation to mitigate overfitting and ensure robustness of our findings.

First, the present population was comparable with prior studies with a median PFS (6 months) and a PFS probability of 2 years (21.9%) [26,39]. In the exploratory univariable survival analysis, several clinicopathological variables emerged as significant predictors of poorer PFS. Specifically, the presence of bone metastases (HR = 1.46, *p* = 0.0439), ≥4 distinct metastatic sites at baseline (HR = 1.66, *p* = 0.0462), and a WHO-PS score of 2 (HR = 2.37, *p* = 0.0015) were associated with inferior outcomes. High tumor burden and bone involvement have been previously linked to a lower response rate to CPI in NSCLC [40,41]. We attribute the adverse impact of WHO-PS = 2 to factors such as a more advanced disease with a greater number of metastases, and, thus, an increased steroid use for burden symptoms [42]. Additionally, a trend towards improved response was observed among patients with PD-L1 TPS ≥ 50% (HR = 0.74, *p* = 0.0825), consistent with the existing literature [35,43,44]. The purpose of collecting these well-known clinicopathological characteristics was to evaluate whether more complex models, incorporating radiomics-based data and advanced survival algorithms, offer added value compared to a reference clinicopathological model, and to determine if they can complement this simpler model.

Secondly, concerning the radiomics-based features, the univariable exploratory survival analysis identified 11 features significantly associated with PFS (and 14 that reached a *p*-value threshold of 0.100), predominantly linked to the largest tumor lesion or the maximum value of RFs across all metastases per patient. However, these features exhibited high correlations among themselves and with certain clinicopathological features, notably, initial staging and the number of metastatic sites, suggesting redundancy in the information they conveyed. Regarding the IPITH metrics, three were selected, all utilizing the Canberra distance. This distance is commonly employed in unsupervised clustering to assess the similarity between different observations, offering several advantages such as simultaneous sensitivity to minor differences and robustness to outliers. Notably, the Canberra-min and the Canberra-mean exhibited a negative correlation with the Canberra-range (rho = −0.86, *p* < 0.0001 and rho = −0.17, *p* = 0.0405, respectively). Consequently, while higher values of Canberra-min and Canberra-mean were generally associated with better PFS outcomes (HR = 0.75, *p* = 0.0006, and HR = 0.81, *p* = 0.0886, respectively), elevated Canberra-range values were logically linked to lower PFS (HR = 1.30, *p* = 0.0049). It must be noted that alternative multisite radiomics-based metrics have been explored in various cancer types. For example, Vargas et al. proposed a method for ovarian cancers using CT-scan imaging, which involved computing local texture maps and per-voxel clustering instead of per-lesion clustering. They also introduced an inter-site heterogeneity matrix based on lesion topography, although this approach may not be directly applicable to NSCLC [45]. Similarly, Zhao et al. recently investigated metastatic NSCLC using ^18^F-FDG positron emission tomography and proposed a meta-histogram approach based on radiomics features from all lesions per patient. However, the use of histograms with only four values, reflecting the median number of tumor lesions per patient, may not be suitable for our cohort [46]. However, none of these works investigated the PFS of MLUAD patients undergoing first-line CPI.

Thirdly, the importance plots of the two best-performing models highlighted the most influential predictor variables contributing to their robust performance. Interestingly, the same RFs were ranked second in importance, with GLSZM_NormalizedZoneSizeNonUniformity from the largest tumor exhibiting significant influence. While IPITH showed limited importance for Deepsurv, it was notable that the Canberra-min and the Canberra-mean significantly contributed to the GBM model. The disparity in the importance rankings of predictor variables between the two models could be attributed to their distinct underlying architectural principles, capturing diverse relationships.

Conceptually, the association between higher Canberra-min and Canberra-mean values and prolonged PFS could imply that an increased dissimilarity between metastatic tumors within a patient, indicating higher inter-site heterogeneity, may correlate with a better prognosis under CPI. Previous research has suggested that the presence of oncogenic drivers leads to genetically homogeneous metastases with lower mutational burden, potentially promoting resistance to CPI by inhibiting T cell recruitment through genetic alterations in tyrosine kinase receptors [5,47,48,49]. Consequently, we could hypothesize that the absence of oncogenic driver alterations, as observed in our cohort, may result in more heterogeneous metastases, contributing to improved outcomes under CPI.

Fourthly, in terms of the final models, although LASSO, RSF, and Deepsurv were anticipated to effectively handle multidimensional correlated data, we observed consistent performance declines when trained on the full input compared to the more streamlined uncorrelated input. Only GBM exhibited performance gains when incorporating all predictor variables. Furthermore, we noted that the stepwise Cox models consistently underperformed compared to other machine-learning algorithms, underscoring the necessity for advanced survival algorithms to improve prognostication in immune-oncology and NSCLC. Similarly encouraging results with Deepsurv have recently been reported in predicting survival outcomes in NSCLC patients undergoing radical radiotherapy based on clinicopathological predictors [50], as well as in forecasting patient outcomes after adjuvant chemotherapy [51]. Moreover, the addition of radiomics-based features, whether in the full input or the uncorrelated input, consistently yielded higher c-indices, underscoring the value of integrating quantitative imaging in onco-immunology, as highlighted in numerous studies [13].

However, while the top-performing models (GBM on full input and Deepsurv on uncorrelated input) significantly outperformed the random model (i.e., the lower bound of their 95% CI exceeded a c-index of 0.50), their performances remained moderate, with c-indices in rCV not surpassing 0.631. Actually, this suggests that radiomics capture only a portion of the prognostic information in MLUAD patients at a macroscopic and global scale. We believe that augmenting these radiomics data with complementary data at the histological scale (such as pathomics approaches) and the molecular scale through gene expression analysis (from tumor samples and liquid biopsies) and tumor microenvironment biomarkers (i.e., features from molecular and immune-histological scales, distinct from the macroscopic radiological scale) is likely to enhance these performances.

The limitations of our study include its retrospective nature, which resulted in missing data that have been previously associated with PFS, such as corrected tumor mutational burden [5,6,52], combinations of STK11, KEAP, and EGFR mutations [6], human leukocyte antigens [5], or tertiary lymphoid structure [7]. The retrospective nature of our study could have also led to bias in the data collection. Second, the population was too small (N = 140) to enable an independent testing cohort to validate the best survival models. However, our methodology prevented the risk of overfitting through the assessment of performances in repeated cross-validation (which could also explain the moderate performances of the best models). We also applied a parsimonious approach to reduce the number of features entered in the modeling and the risk of false discoveries, without failing to capture the relevant relationships between radiomics and sensitivity to CPI with too stringent correction for multiple comparisons in an original exploratory biomarker study. Third, some tumor lesions were not analyzable with radiomics due to their too-small size or complex shape, such as bone metastases without extra-osseous spreading, pleural effusion, or meningeal carcinomatosis. Thus, we believe that combining radiomics-based features, which capture intra- and inter-tumoral heterogeneity and radiophenotypes, with descriptors of metastatic spread, such as the number and the presence of metastases in specific organs, would currently be an effective way to obtain a comprehensive picture of the disease in patients with MLUAD. Fourth, the interaction between intra- and inter-tumoral heterogeneity, which we attempted to capture individually using single-site radiomics, multi-site radiomics, and IPITH, remains complex to understand, and the underlying histological, immune, and gene-expression patterns have yet to be fully elucidated.

## 5. Conclusions

In summary, this exploratory study demonstrates the synergistic potential of (i) incorporating clinicopathological features, (ii) radiomics-based data to capture the radiophenotypes of both individual tumors and metastatic lesions within the same patient, through metrics designed to measure their similarity or divergence, and (iii) employing advanced machine-learning models capable of handling complex linear and non-linear relationships within multidimensional dataset. While the performances of the best models (i.e., Deepsurv trained on the uncorrelated input dataset, and GBM trained on the full input dataset) appeared moderate, they notably outperformed both the random model and the traditional Cox models. These findings underscore the relevance of integrating single- and multisite radiomics data and advanced shallow and deep-learning algorithms into the field of onco-immunology and pave the way for the development of more comprehensive and accurate radiogenomics biomarker signatures for predicting the response to CPI.

## Figures and Tables

**Table 2:** Univariable survival analysis for the clinical and pathological initial variables.

Characteristics	No. at Risk	No. of Events	PFS Probability at 2 Years (95%CI)	Log-Rank *p*-Value	Univariable HR (95%CI)	*p*-Value
**Age at diagnosis**						
<70 years	103	85	31.07 (23.3–41.42)	0.6447	reference	-
≥70 years	37	31	29.73 (18.12–48.79)		1.11 (0.73–1.67)	0.6327
**Sex**						
Women	51	45	25.49 (15.94–40.75)	0.4047	reference	-
Men	88	70	34.09 (25.5–45.58)		0.86 (0.59–1.25)	0.4262
**WHO-PS**						
PS = 0	38	33	28.95 (17.59–47.64)	0.0003 ***	reference	-
PS = 1	77	59	38.96 (29.46–51.53)		0.92 (0.6–1.41)	0.6921
PS = 2	25	24	8 (2.12–30.23)		2.37 (1.39–4.05)	0.0015 **
**Tobacco addiction**						
Never smoker	6	6	0 (NA–NA)	0.3477	reference	-
Active smoker	67	52	35.82 (26–49.36)		0.77 (0.33–1.8)	0.5464
Former smoker	67	58	28.36 (19.38–41.49)		1.01 (0.43–2.35)	0.9806
**Initial staging**						
IIIB-IVA	36	28	44.44 (30.85–64.04)	0.0829	reference	-
IVB	104	88	25.96 (18.77–35.92)		1.46 (0.95–2.23)	***0.0844***
**PDL1**						
0%	43	38	27.91 (17.26–45.12)	0.0779	reference	-
1–49%	35	32	22.86 (12.44–42.01)		0.81 (0.6–1.1)	0.1826
50–100%	62	46	37.1 (26.83–51.3)		0.74 (0.53–1.04)	***0.0825***
**No. of altered genes on routine screening**						
0	29	25	24.14 (12.66–46.02)	0.7005	reference	-
1	73	59	31.51 (22.47–44.19)		0.87 (0.54–1.38)	0.5484
≥2	38	32	34.21 (22.01–53.17)		0.8 (0.47–1.35)	0.4032
**TP53 alteration**						
No or non-contributive	85	70	29.41 (21.16–40.88)	0.8992	reference	-
Yes	55	46	32.73 (22.41–47.8)		0.97 (0.67–1.41)	0.8890
**KRAS alteration**						
No or non-contributive	73	62	26.03 (17.68–38.32)	0.2002	reference	-
Yes	67	54	35.82 (26–49.36)		0.79 (0.55–1.14)	0.2003
**No. of distinct metastatic sites**						
1	35	27	37.14 (24.14–57.15)	0.0587	reference	-
2	36	28	41.67 (28.31–61.33)		0.91 (0.54–1.55)	0.7304
3	29	25	24.14 (12.66–46.02)		1.41 (0.82–2.43)	0.2183
≥4	40	36	20 (10.76–37.17)		1.66 (1.01–2.74)	0.0462 *
**Bone metastasis**						
No	69	53	37.68 (27.82–51.04)	0.0427 *	reference	-
Yes	71	63	23.94 (15.82–36.24)		1.46 (1.01–2.11)	0.0439 *
**Brain metastasis**						
No	108	87	32.41 (24.68–42.55)	0.1638	reference	-
Yes	32	29	25 (13.72–45.56)		1.35 (0.88–2.06)	0.1692
**Liver metastasis**						
No	112	92	33.04 (25.38–43)	0.4336	reference	-
Yes	28	24	21.43 (10.54–43.55)		1.2 (0.76–1.88)	0.4351
**First-line treatment**						
CPI + Chemotherapy	110	93	30 (22.55–39.91)	0.2766	reference	-
CPI	30	23	33.33 (20.1–55.29)		0.78 (0.49–1.23)	0.2812

NOTE—Abbreviations: 95%CI, 95% confidence interval; CPI, checkpoint inhibitor; HR, hazard ratio; no., number; PFS, progression free survival; WHO-PS, World Health Organization performance status. *: *p* < 0.05, **: *p* < 0.005, ***: *p* < 0.001. Significant results are in bold. Variables associated with PFS with *p*-value between 0.05 and 0.100 are in italic bold (and were also subsequently included in the multivariable machine-learning analysis).

**Table 3:** Summary of the univariable survival analysis of the radiomics-based features in the entire population.

Type of Radiomics	Name of Radiomics-Based Feature (IBSI Reference Number)	HR (95%CI)	Univariable Cox *p*-Value
**Largest**	GLSZM_NormalisedZoneSizeNonUniformity (IBSI: VB3A)	1.46 (1.11–1.94)	0.0076 *
	GLSZM_SmallZoneEmphasis(IBSI: 5QRC)	1.44 (1.09–1.9)	0.0092 *
	GLSZM_ZonePercentage (IBSI: P30P)	1.25 (1–1.56)	0.0495 *
**Average**	GLSZM_NormalisedZoneSizeNonUniformity (IBSI: VB3A)	1.25 (0.97–1.61)	0.0887
	GLSZM_SmallZoneEmphasis (IBSI: 5QRC)	1.25 (0.96–1.61)	0.0921
**Minimum**	GLRLM_LongRunsEmphasis (IBSI: W4KF)	0.65 (0.43–0.98)	0.0417 *
	GLSZM_ZonePercentage (IBSI: P30P)	1.24 (1.01–1.51)	0.0384 *
**Maximum**	GLSZM_SmallZoneEmphasis (IBSI: 5QRC)	1.21 (1.01–1.44)	0.0403 *
	GLRLM_RunPercentage (IBSI: 9ZK5)	1.4 (1.01–1.93)	0.0421 *
	GLRLM_ShortRunsEmphasis (IBSI: 22OV)	1.38 (1.01–1.9)	0.0437 *
	GLSZM_NormalisedZoneSizeNonUniformity (IBSI: VB3A)	1.18 (1.01–1.39)	0.0445 *
**IPITH**	Canberra-min	0.75 (0.63–0.88)	0.0006 ***
	Canberra-range	1.30 (1.08–1.57)	0.0049 **
	Canberra-mean	0.81 (0.64–1.03)	0.0886

NOTE—Abbreviations: 95%CI, 95% confidence interval; HR, hazard ratio; IBSI, imaging biomarker standardization initiative; IPITH, intra-patient inter tumor heterogeneity metrics. *: *p* < 0.05, **: *p* < 0.005, ***: *p* < 0.001. Significant results are in bold.

**Table 4:** Performances of the models to predict progression-free survival depending on the input variables, according to concordance index in 100-times repeated 5-fold cross-validation.

Algorithms	Clinical Input		Full Input		Uncorrelated Input	
	rCV C-index	Hyperparameters	rCV C-index	Hyperparameters	rCV C-index	Hyperparameters
**Stepwise Cox Regression**	0.566 (0.525–0.601)	-	0.560 (0.517–0.606)	-	0.570 (0.538–0.602)	-
**LASSO Cox Regression**	0.583 (0.549–0.613)	λ = 0.019	0.573 (0.535–0.613)	λ = 0.058	0.582 (0.554–0.616)	λ = 0.011
**Random Survival Forests**	0.599 (0.581–0.616)	mtry = 1, nodesize = 22	0.593 (0.567–0.618)	mtry = 1, nodesize = 20	0.602 (0.576–0.626)	mtry = 1, nodesize = 22
**Gradient Boosted Model**	0.560 (0.527–0.589)	shrinkage = 0.05, interaction depth = 2, MNOTN = 8	**0.603 (0.557–0.646)**	shrinkage = 0.01, interaction depth = 4, MNOTN = 11	0.594 (0.546–0.634)	shrinkage = 0.095, interaction depth = 1, MNOTN = 11
**Deepsurv**	**0.622 (0.602–0.647)**	no. layers = 3, no. nodes = 14, adam optimizer, ReLU activation	0.613 (0.581–0.634)	no. layers = 2, no. nodes = 15, adam optimizer, SELU activation	**0.631 (0.625–0.647)**	no. layers = 1, no. nodes = 15, adam optimizer, ReLU activation

NOTE—Abbreviations: c-index, concordance index; LASSO, least absolute shrinkage and selection operator; MNOTN, minimal number of observations in the terminal nodes of the trees; no., number; rCV, repeated cross-validation; ReLU, rectified linear units; SELU, scaled exponential linear units. C-indices in bold correspond to the highest c-index value for each type of input.

## Data Availability

The radiomics datasets and raw MRIs used and/or analyzed during the current study are available from the corresponding author on reasonable request. Any additional results can be obtained from the corresponding author. The underlying code for this study is not publicly available but may be made available to qualified researchers on reasonable request from the corresponding author. The R studio environment and the packages with their versions used for the analyses are detailed in Supplementary Table S3.

## Supplementary Materials

The following supporting information can be downloaded at: https://www.mdpi.com/article/10.3390/cancers16132491/s1, Table S1: Reproducible radiomics features over two 3D segmentations; Table S2: Distance metrics used to calculate the similarity or dissimilarity between each pair of radiomics target lesions found in a patient according to their radiophenotypes as evaluated with the reproducible radiomics features; Table S3: Software, packages, versions and functions used for the statistical analyses; Table S4: Tuning grids used for the training of survival algorithms; Table S5: Performances of usual stepwise Cox regression models on the entire cohort with the three types of input.
