# Supplementary material for: Enhancing Immunotherapy Response Prediction in Metastatic Lung Adenocarcinoma: Leveraging Shallow and Deep Learning with CT-Based Radiomics across Single and Multiple Tumor Sites
Source: Cancers (Basel). 2024 Jul 8;16(13):2491. doi: 10.3390/cancers16132491 (PMC11240700; doi:10.3390/cancers16132491)
Supplement: Supplementary file 1 [file cancers-16-02491-s001.zip › cancers-3018579-supplementary.pdf]

Supplementary Materials

Supplementary Table S1. Reproducible radiomics features over two 3D segmentations.

Reproducible radiomics features	ICC (95%CI)
MORPHOLOGICAL_ApproximateVolume_IBSI_YEKZ	0.954 (0.431 - 0.987)
MORPHOLOGICAL_Compacity_IBSI_No	0.916 (-0.009 - 0.981)
MORPHOLOGICAL_Compactness1_IBSI_SKGS	0.964 (0.841 - 0.985)
MORPHOLOGICAL_Compactness2_IBSI_BQWJ	0.965 (0.862 - 0.986)
MORPHOLOGICAL_IntegratedIntensity_IBSI_99N0	0.953 (0.511 - 0.986)
MORPHOLOGICAL_Maximum3DDiameter_IBSI_L0JK	0.995 (0.677 - 0.999)
MORPHOLOGICAL_Sphericity_IBSI_QCFX	0.96 (0.845 - 0.983)
MORPHOLOGICAL_SurfaceArea_IBSI_C0JK	0.968 (0.286 - 0.992)
MORPHOLOGICAL_Volume_IBSI_RNU0	0.954 (0.427 - 0.987)
MORPHOLOGICAL_voxelsCounting_IBSI_No	0.954 (0.431 - 0.987)
INTENSITY_BASED_10thPercentileIBSI_QG58	0.873 (0.801 - 0.918)
INTENSITY_BASED_25thPercentileIBSI_No	0.948 (0.921 - 0.966)
INTENSITY_BASED_50thPercentileIBSI_Y12H	0.987 (0.981 - 0.992)
INTENSITY_BASED_75thPercentileIBSI_No	0.987 (0.98 - 0.992)
INTENSITY_BASED_90thPercentileIBSI_8DWT	0.99 (0.981 - 0.994)
INTENSITY_BASED_EnergyIBSI_N8CA	0.951 (0.569 - 0.984)
INTENSITY_BASED_MeanIBSI_Q4LE	0.966 (0.948 - 0.977)
INTENSITY_BASED_MedianIBSI_Y12H	0.987 (0.981 - 0.992)
INTENSITY_BASED_RootMeanSquareIBSI_5ZWQ	0.948 (0.921 - 0.966)
INTENSITY_HISTOGRAM_AreaUnderCurveCshIBSI_No	0.978 (0.967 - 0.985)
INTENSITY_HISTOGRAM_IntensityHistogram10thPercentileIBSI_GPMT	0.905 (0.84 - 0.941)
INTENSITY_HISTOGRAM_IntensityHistogram25thPercentileIBSI_No	0.948 (0.92 - 0.966)
INTENSITY_HISTOGRAM_IntensityHistogram50thPercentileIBSI_No	0.987 (0.98 - 0.991)
INTENSITY_HISTOGRAM_IntensityHistogram75thPercentileIBSI_No	0.984 (0.975 - 0.989)
INTENSITY_HISTOGRAM_IntensityHistogram90thPercentileIBSI_OZ0C	0.988 (0.979 - 0.992)
INTENSITY_HISTOGRAM_IntensityHistogramEntropyLog10IBSI_No	0.897 (0.076 - 0.971)
INTENSITY_HISTOGRAM_IntensityHistogramEntropyLog2IBSI_TLU2	0.897 (0.076 - 0.971)
INTENSITY_HISTOGRAM_IntensityHistogramMeanAbsoluteDeviationIBSI_D2ZX	0.873 (0.636 - 0.941)
INTENSITY_HISTOGRAM_IntensityHistogramMeanIBSI_X6K6	0.978 (0.967 - 0.985)
INTENSITY_HISTOGRAM_IntensityHistogramMedianAbsoluteDeviationIBSI_4RNL	0.874 (0.618 - 0.943)
INTENSITY_HISTOGRAM_IntensityHistogramMedianIBSI_WIFQ	0.987 (0.98 - 0.991)
INTENSITY_HISTOGRAM_IntensityHistogramStandardDeviationIBSI_No	0.865 (0.384 - 0.95)
INTENSITY_HISTOGRAM_MaximumHistogramGradientGreyLevelIBSI_8E6O	0.967 (0.952 - 0.978)
INTENSITY_HISTOGRAM_MaximumHistogramGradientIBSI_12CE	0.982 (0.827 - 0.994)
INTENSITY_HISTOGRAM_MinimumHistogramGradientIBSI_VQB3	0.93 (0.817 - 0.966)
INTENSITY_HISTOGRAM_UniformityIBSI_BJ5W	0.905 (0.316 - 0.969)
GLCM_AngularSecondMoment_IBSI_8ZQL	0.902 (0.504 - 0.964)
GLCM_Autocorrelation_IBSI_QWB0	0.988 (0.982 - 0.992)
GLCM_Correlation_IBSI_NI2N	0.938 (0.909 - 0.958)
GLCM_DifferenceAverage_IBSI_TF7R	0.881 (0.628 - 0.947)
GLCM_DifferenceEntropy_IBSI_NTRS	0.905 (0.143 - 0.973)
GLCM_Dissimilarity_IBSI_8S9J	0.881 (0.628 - 0.947)
GLCM_InverseDifference_IBSI_IB1Z	0.933 (0.401 - 0.979)
GLCM_InverseDifferenceMoment_IBSI_WF0Z	0.937 (0.406 - 0.981)
GLCM_JointAverage_IBSI_60VM	0.984 (0.976 - 0.989)
GLCM_JointEntropyLog10_IBSI_No	0.905 (0.143 - 0.973)
GLCM_JointEntropyLog2_IBSI_TU9B	0.905 (0.143 - 0.973)
GLCM_JointMaximum_IBSI_GYBY	0.904 (0.654 - 0.959)
GLCM_SumAverage_IBSI_ZGXS	0.984 (0.976 - 0.989)
GLRLM_GreyLevelNonUniformity_IBSI_R5YN	0.975 (0.651 - 0.993)
GLRLM_HighGreyLevelRunEmphasis_IBSI_G3QZ	0.987 (0.98 - 0.991)

GLRLM_LongRunHighGreyLevelEmphasis_IBSI_3KUM	0.988 (0.982 - 0.992)
GLRLM_LongRunsEmphasis_IBSI_W4KF	0.974 (0.951 - 0.985)
GLRLM_RunLengthNonUniformity_IBSI_W92Y	0.953 (0.443 - 0.986)
GLRLM_RunPercentage_IBSI_9ZK5	0.977 (0.955 - 0.987)
GLRLM_ShortRunHighGreyLevelEmphasis_IBSI_GD3A	0.986 (0.979 - 0.991)
GLRLM_ShortRunsEmphasis_IBSI_22OV	0.975 (0.944 - 0.987)
GLSZM_GreyLevelNonUniformity_IBSI_JNSA	0.974 (0.724 - 0.992)
GLSZM_HighGrayLevelZoneEmphasis_IBSI_5GN9	0.978 (0.961 - 0.987)
GLSZM_LargeZoneEmphasis_IBSI_48P8	0.992 (0.988 - 0.995)
GLSZM_LargeZoneHighGreyLevelEmphasis_IBSI_J17V	0.99 (0.985 - 0.993)
GLSZM_NormalisedZoneSizeNonUniformity_IBSI_VB3A	0.897 (0.622 - 0.957)
GLSZM_SmallZoneEmphasis_IBSI_5QRC	0.889 (0.593 - 0.954)
GLSZM_SmallZoneHighGreyLevelEmphasis_IBSI_HW1V	0.98 (0.971 - 0.987)
GLSZM_ZonePercentage_IBSI_P30P	0.978 (0.955 - 0.988)
GLSZM_ZoneSizeNonUniformity_IBSI_4JP3	0.94 (0.459 - 0.981)
GLSZM_ZoneSizeVariance_IBSI_3NSA	0.992 (0.988 - 0.995)
NGTDM_Busyness_IBSI_NQ30	0.959 (0.939 - 0.973)

NOTE.- Abbreviations: 95%CI: 95% confidence interval, GLCM: gray level co-occurrence matrix, GLRLM: gray level run length, GLSZM: gray level size zone matrix, ICC: intra-class correlation coefficient, NGTDM: neighborhood gray tone difference matrix.
The names are followed by the imaging biomarker standardization initiative (IBSI) identifier.

Supplementary Table S2. Distance metrics used to calculate the similarity or dissimilarity between each pair of radiomics target lesions found in a patient according to their radiophenotypes as evaluated with the reproducible radiomics features.

Let's name:
- N: the number of elements of P and Q, which corresponds to the number of reproducible radiomics features
- P: a tumor lesion from a given patient described by N radiomics features
- Q: a tumor lesion from the same given patient also described by N radiomics features

Both P and Q tumors can be represented in a radiomics space of N dimensions in which their distance can be calculated according to the following formula:

Distance	Principle	Pros	Cons	Formula
**Euclidean**	Measures the straight line distance between two points. Most commonly used distance measure between two points in a multidimensional space.	- Simple and easy to understand and calculate<br>- Intuitive<br>- Computationally efficient<br>- Widely applicable	- Sensitive to scale: variables with larger ranges or magnitudes may dominate the distance calculations, leading to biased results.<br>- Assumes linear relationships between the variables of the dataset<br>- Limited to Euclidean space<br>- Vulnerable to outliers	$= \sqrt{\sum_{i=1}^{N} \lvert P_i - Q_i \rvert^2}$
**Minkowski**	Generalization of Euclidean and Manhattan distances	- Flexibility by adjusting the p parameter to fit the characteristics of the dataset.<br>- Versatile	- Sensitivity to the choice of p (if inappropriate, can lead to suboptimal measurement)<br>- Computational complexity and expensive<br>- Vulnerable to outliers	$= (\sum_{i=1}^{N} \lvert P_i - Q_i \rvert^p)^{1/p}$
**Chebyshev**	Measures the maximum difference between corresponding components of two vectors.	- Simple interpretation, straightforward measure of dissimilarity<br>- Robustness to outliers, handle well noisy data<br>- Applicability to irregularly shaped grouping<br>- Flexibility with numerical and categorical data	- Simple interpretation, straightforward measure of dissimilarity<br>- Robustness to outliers, handle well noisy data<br>- Applicability to irregularly shaped grouping of points<br>- Flexibility with numerical and categorical data	$= \max \lvert P_i - Q_i \rvert$

**Spearman**	Non-parametric, rank-based method. Measures the correlation between two variables in a dataset based on their rank order.	- Robustness to outlier<br>- Applicable to ordinal data<br>- Can capture monotonic relationships (i.e., even if the relationship is not linear)<br>- Simple interpretation	- Loss of information and sensitivity as only the ranks are considered<br>- Computational complexity and expensive<br>- Sensitivity to ties, when tied ranks, can lead to inaccuracy	$\rho(P,Q) = 1 - \frac{6 \times \sum_{i=1}^{N}(rank(P_i) - rank(Q_i))^2}{N \times (N^2 - 1)}$<br>$Spearman\ Distance = \frac{1}{2} \times (1 - \rho(P,Q))$
**Jaccard**	Belongs to the inner product family.<br>Measures the similarity between two sets by comparing intersection and union. Ignores the magnitude of sets.	- Robust against variations in dataset size<br>Intuitive interpretation<br>- Computationally efficient<br>- Robust to scale	- Loss of information<br>- Simplification of the dataset	$= 1 - \frac{\sum_{i=1}^{N} P_i \times Q_i}{\sum_{i=1}^{N} {P_i}^2 + \sum_{i=1}^{N} {Q_i}^2 + \sum_{i=1}^{N} P_i \times Q_i}$
**Canberra**		- Sensitivity to differences.<br>- Can effectively capture small differences between data points.<br>- Robustness to outliers<br>- Suitable for sparse data	- Sensitivity to zero values<br>- Assumes equal importance for all attributes	$= \frac{\sum_{i=1}^{N} \lvert P_i - Q_i \rvert}{\lvert P_i \rvert + \lvert Q_i \rvert}$

Supplementary Table S3. Software, packages, versions and functions used for the statistical analyses.

Softwares and packages	Source	Version	Functions
R	https://www.r-project.org/	v4.1.0 and v4.3.3	-
Rstudio	http://www.rstudio.com/products/rstudio/download/	v1.4.1106	-
**MRI post-processing**			
"ANTsR" R package	https://github.com/ANTsX/ANTsR	v0.5.6.0.0	*(required for ANTsRCore)*
"ANTsRCore" R package	https://github.com/ANTsX/ANTsRCore	v0.7.4	denoiseImage, antsImageRead, antsImageWrite
"oro.dicom" R package	https://github.com/cran/oro.dicom	v0.5.3	readDICOM, dicom2nifti
"oro.nifti" R package	https://github.com/neuroconductor/oro.nifti	v0.11.0	writeNIfTI
"extrantsr" R package	https://github.com/muschellij2/extrantsr	v3.9.1	resample_image
"irr" R package	https://github.com/cran/irr	v0.84.1	icc
**Data manipulation**			
"openxlsx" R package	https://github.com/cran/openxlsx	v4.2.4	read.xlsx, write.xlsx
"base" R package	https://github.com/SurajGupta/r-source/blob/master/src/library/base/R/	v4.1.0	list.files, table, min, max, mean, median, sd, quantile, summary, scale, expand.grid, unique, length, round, paste
"boot" R package	https://github.com/cran/boot	v1.3-28	boot, boot.ci
"dplyr" R package	https://github.com/tidyverse/dplyr	v1.1.2	filter, select, arrange, summarize, distinct, top_n, mutate, group_by, bind_rows, bind_cols, left_join
"purrr" R package	https://github.com/tidyverse/purrr	v0.3.4	map, map2, pmap, safely
"stringr" R package	https://github.com/tidyverse/stringr	v1.5.0	str_remove_all
"lubridate" R package	https://github.com/tidyverse/lubridate	v1.7.10	ymd
"forcats" R package	https://github.com/tidyverse/forcats	v0.5.1	case_when, fct_reorder
"amap" R package	https://github.com/cran/amap	V0.8-18	Dist
"Philentropy" R package	https://github.com/drostlab/philentropy	v0.7.0	distance
**Data visualization**			
"survminer" R package	https://github.com/kassambara/survminer	v0.4.9	ggsurvplot
"ggplot2" R package	https://github.com/tidyverse/ggplot2	v3.4.2	ggplot, ggsave
"cowplot" R package	https://github.com/wilkelab/cowplot	v1.1.1	plot_grid
"corrplot" R package	https://github.com/cran/corrplot/blob/master/R/corrplot.R	v0.92	corrplot
**Statistical analyses**			
"stats" R package	https://github.com/SurajGupta/r-source/blob/master/src/library/stats/R/	v4.1.0	chisq.test, table, shapiro.test, cor.test, wilcox.test, t.test, kruskal.test, cor.test, relevel, ordered, predict
"rms" R package	https://github.com/cran/rms	v6.2-0	cph
"MASS" R package	https://github.com/cran/MASS	v7.3-54	stepAIC
"caret" R package	https://github.com/topepo/caret	v6.0-88	createMultiFolds, dummyVars
Hmisc	https://github.com/harrelfe/Hmisc	v4.5-0	rcorr.cens
"survMetrics" R package	https://github.com/cran/SurvMetrics/	v0.5.0	Brier, IBS
"survival" R package	https://github.com/therneau/survival	v3.2-11	coxph, Surv, survdiff, survfit, summary, predict, basehaz
"satpred" R package	https://github.com/CYGUBICKO/satpred	v1.0	get_varimp

"gbm" R package	https://github.com/gbm-developers/gbm	v2.1.8.1	gbm, summary.gbm, basehaz.gbm
"glmnet" R package	https://github.com/cran/glmnet	v4.1-1	glmnet
"randomForestSRC" R package	https://github.com/cran/randomForestSRC	v3.2.1	rfsrc, predict.rfsrc, get.cindex
"survivalmodels" R package	https://github.com/RaphaelS1/survivalmodels	v0.1.191	deepsurv, cindex, set_seed, predict.pycox

Supplementary Table S4. Tuning grids used for the training of survival algorithms.

Algorithm	Hyperparameters	Investigated values during training
**Stpewise Cox regression**	none	-
**LASSO Cox regression**	λ	1.367346e-01, 1.245875e-01, 1.135195e-01, 1.034347e-01, 9.424589e-02, 8.587334e-02, 7.824459e-02, 7.129356e-02, 6.496003e-02, 5.918916e-02, 5.393096e-02, 4.913988e-02, 4.477443e-02, 4.079679e-02, 3.717252e-02, 3.387022e-02, 3.086128e-02, 2.811965e-02, 2.562158e-02, 2.334543e-02, 2.127148e-02, 1.938178e-02, 1.765996e-02, 1.609110e-02, 1.466161e-02, 1.335911e-02, 1.217232e-02, 1.109097e-02, 1.010568e-02, 9.207917e-03, 8.389911e-03, 7.644574e-03, 6.965451e-03, 6.346660e-03, 5.782840e-03, 5.269109e-03, 4.801016e-03, 4.374507e-03, 3.985887e-03, 3.631792e-03, 3.309154e-03, 3.015178e-03, 2.747318e-03, 2.503253e-03, 2.280871e-03, 2.078245e-03, 1.893619e-03, 1.725396e-03, 1.572116e-03, 1.432454e-03, 1.305198e-03, 1.189248e-03, 1.083599e-03, 9.873348e-04, 8.996227e-04, 8.197027e-04, 7.468825e-04, 6.805315e-04, 6.200750e-04, 5.649892e-04, 5.147972e-04, 4.690640e-04, 4.273936e-04, 3.894252e-04, 3.548297e-04, 3.233076e-04, 2.945859e-04, 2.684157e-04, 2.445704e-04, 2.228434e-04, 2.030466e-04, 1.850085e-04, 1.685729e-04, 1.535973e-04, 1.399522e-04, 1.275192e-04, 1.161907e-04, 1.058687e-04, 9.646359e-05, 8.789403e-05, 8.008577e-05, 7.297117e-05, 6.648861e-05, 6.058194e-05, 5.520001e-05, 5.029619e-05, 4.582802e-05, 4.175678e-05, 3.804723e-05, 3.466722e-05, 3.158748e-05, 2.878133e-05, 2.622448e-05, 2.389477e-05, 2.177202e-05, 1.983786e-05, 1.807552e-05, 1.646974e-05, 1.500661e-05, 1.367346e-05
**Random survival forests**	mtry	from 1 to 10 with an increment of 1
	nodesize	from 5 to 25 with an increment of 1
**Gradient boosted machine**	shrinkage	from 0.01 to 0.2 with an increment of 0.01
	interaction depth	from 1 to 20 with an increment of 1
	minimal no. of observations in the terminal nodes of the trees	from 5 to 45 with an increment of 5
**Deepsurv**	no. of layers	1,2,3,4
	no. of nodes	from 4 to 20 with an increment of 1
	optimizer	adam, sgd
	activation function	ReLU, SELU

Supplementary Table S5. Performances of usual stepwise Cox regression models on the entire cohort with the three types of input.

Predictor variables entered in the modeling	Clinicopathological input		Full input		Uncorrelated input	
	HR (95%CI)	P-value	HR (95%CI)	P-value	HR (95%CI)	P-value
WHO-PS = 1 (ref.: 0)	0.83 (0.53 - 1.29)	0.40775	0.76 (0.48 - 1.21)	0.2454	0.78 (0.50 - 1.21)	0.2639
WHO-PS = 2 (ref.: 0)	2.16 (1.25 - 3.71)	**0.0056****	1.72 (0.96 - 3.06)	0.0676	1.83 (1.04 - 3.22)	**0.0372***
Initial staging = IVB (ref: IIIB-IVA)	1.46 (0.94 - 2.28)	0.0910	*step 9*		*step 3*	
PD-L1 = 1-49% (ref.: 0%)	*step 2*		*step 2*		*step 2*	
PD-L1 = 50-100% (ref.: 0%)	*step 2*		*step 2*		*step 2*	
No. of metastatic sites = 2 (ref.: 1)	*step 1*		*step 1*		*step 1*	
No. of metastatic sites = 3 (ref.: 1)	*step 1*		*step 1*		*step 1*	
No. of metastatic sites = 4 (ref.: 1)	*step 1*		*step 1*		*step 1*	
Bone metastases (ref.: no)	*step 3*		1.41 (0.95 - 2.10)	0.0868	1.45 (0.99 - 2.13)	0.0595
GLSZM_NormalisedZoneSizeNonUniformity.largest	-	-	step 4		1.35 (1.03 - 1.78)	**0.0325***
GLSZM_SmallZoneEmphasis.largest	-	-	1.66 1.10 - 2.51	**0.0156***	-	-
GLSZM_ZonePercentage.largest	-	-	*step 6*		-	-
GLSZM_NormalisedZoneSizeNonUniformity.average	-	-	0.60 (0.33 - 1.08)	0.0876	-	-
GLSZM_SmallZoneEmphasis.average	-	-	*step 11*		-	-
GLRLM_LongRunsEmphasis.minimum	-	-	*step 3*		-	-
GLSZM_ZonePercentage.maximum	-	-	1.42 (1.01 - 2.01)	**0.0486***	-	-
GLSZM_SmallZoneEmphasis.maximum	-	-	*step 13*		-	-
GLRLM_RunPercentage.maximum	-	-	*step 12*		-	-
GLRLM_ShortRunsEmphasis.maximum	-	-	*step 5*		-	-
GLSZM_NormalisedZoneSizeNonUniformity.maximum	-	-	*step 10*		-	-
Canberra-min	-	-	0.77 (0.65 - 0.91)	**0.0027****	-	-
Canberra-range	-	-	*step 8*		-	-
Canberra-mean	-	-	*step 7*		0.82 (0.66 - 1.02)	0.0810
**Change in AIC**	AIC from 983.38 to 977.56		AIC from 994.76 to 970.95		AIC from 980.48 to 973.54	

NOTE.- Abbreviations: 95%CI: 95% confidence interval, AIC: Akaike information criteria, HR: hazard ratio.
*: P<0.05, **: P<0.005. Significant results are in bold. Step i means that the variable has been removed at the i-th step of the stepwise selection process of the Cox regression.
